# Hyperphosphatemia and Cardiovascular Disease

**DOI:** 10.3389/fcell.2021.644363

**Published:** 2021-03-04

**Authors:** Chao Zhou, Zhengyu Shi, Nan Ouyang, Xiongzhong Ruan

**Affiliations:** ^1^Department of Cardiology, The First Affiliated Hospital of Chongqing Medical University, Chongqing, China; ^2^Department of Nephrology, The First Affiliated Hospital of Chongqing Medical University, Chongqing, China; ^3^John Moorhead Research Laboratory, Centre for Nephrology, University College London (UCL) Medical School, London, United Kingdom; ^4^Centre for Lipid Research and Key Laboratory of Molecular Biology for Infectious Diseases (Ministry of Education), Institute for Viral Hepatitis, Department of Infectious Diseases, The Second Affiliated Hospital, Chongqing Medical University, Chongqing, China

**Keywords:** phosphate, cardiovascular disease, vascular calcification, cardiac valvular calcification, atherosclerosis, left ventricular hypertrophy, myocardial fibrosis, hypertension

## Abstract

Hyperphosphatemia or even serum phosphate levels within the “normal laboratory range” are highly associated with increased cardiovascular disease risk and mortality in the general population and patients suffering from chronic kidney disease (CKD). As the kidney function declines, serum phosphate levels rise and subsequently induce the development of hypertension, vascular calcification, cardiac valvular calcification, atherosclerosis, left ventricular hypertrophy and myocardial fibrosis by distinct mechanisms. Therefore, phosphate is considered as a promising therapeutic target to improve the cardiovascular outcome in CKD patients. The current therapeutic strategies are based on dietary and pharmacological reduction of serum phosphate levels to prevent hyperphosphatemia in CKD patients. Large randomized clinical trials with hard endpoints are urgently needed to establish a causal relationship between phosphate excess and cardiovascular disease (CVD) and to determine if lowering serum phosphate constitutes an effective intervention for the prevention and treatment of CVD.

## Introduction

High serum phosphate concentrations associate with cardiovascular disease (CVD) risk in both the general population and chronic kidney disease (CKD) patients (Lim et al., 2015; Reiss et al., 2018). Serum phosphate levels are tightly regulated in healthy individuals through several mechanisms including dietary absorption, bone flux and renal excretion. Hyperphosphatemia occurs due to a decreasing glomerular filtration rate (GFR) and is known to induce hypertension, vascular calcification, cardiac valvular calcification, atherosclerosis, left ventricular hypertrophy, and myocardial fibrosis (Chronic Kidney, Disease Prognosis, Consortium, Matsushita et al., 2010; Sarnak et al., 2019). In this review, we summarize the current knowledge of the roles of phosphate homeostasis in health and CKD conditions, as well as the contribution of phosphate to CVD. Moreover, we discuss therapeutic strategies for lowering serum phosphate level and how this affects the cardiovascular outcome of CKD patients.

## Biological Characteristics of Phosphate

Phosphorus is an essential element of the human body. Organic phosphorus mainly exists in nucleic acid, phospholipid and high-energy phosphate compounds (Michigami et al., 2018). Phosphorus is one of the basic components of human genetic material nucleic acid, participating in genetic metabolism, growth, and development (Kornberg, 1979). The phospholipid is the main lipid component on the cell membrane, maintaining the integrity and permeability of membrane (Tero et al., 2017). High energy phosphate compounds such as adenosine triphosphate (ATP) are key substances of energy metabolism (Müller et al., 2017). Inorganic phosphorus exists in the form of inorganic phosphate ion H2PO4- or HPO42-. In the physiological pH state, 80% inorganic phosphate ion in the serum is HPO42- and 20% is H2PO4-. About 10% inorganic phosphorus exists in serum as soluble phosphate, which participates in the phosphorylation and dephosphorylation of various signal transduction proteins. Phosphate buffering also participates in the regulation of acid-base balance *in vivo*. A total of 90% inorganic phosphorus exists in bones and teeth in insoluble hydroxyapatite, forming the skeleton of the body. The body contains about 600–900 g phosphorus, accounting for about 1% of body weight. Serum phosphorus refers to the concentration of serum inorganic phosphate, which is 3–4.5 mg/dL in normal adults. Hyperphosphatemia is defined as plasma phosphate >4.5 mg/dL due to disease or excessive intake. Excessive phosphate content not only causes hypocalcemia, hyperparathyroidism and metabolic bone disease but also is closely related to adverse cardiovascular outcomes (Angelova et al., 2016; Chande and Bergwitz, 2018).

## Metabolic Regulation of Phosphate

The organs involved in phosphate regulation include intestine, bone, kidney, and parathyroid gland. The daily intake of dietary phosphate is about 1.0–1.5 g, which is absorbed by the intestinal epithelial cell type II sodium-dependent phosphate co-transporter (Npt)-2a, 2b, and 2c (Masuda et al., 2020). The phosphate absorbed from the intestine is mainly excreted through the kidney. About 80% of the phosphate is reabsorbed through Npt- 2a and 2c in the proximal renal tubular epithelium (Michigami et al., 2018). The key endocrine hormonal regulators of phosphate metabolism include fibroblast growth factor-23 (FGF-23), 1,25-dihydroxyvitamin D (calcitriol), and parathyroid hormone (PTH). FGF-23 is the first discovered bone-derived endocrine hormone, contains a 32 kd peptide chain of 251 amino acids and belongs to the FGF superfamily. Phosphate excess can directly or indirectly stimulate osteoblasts to secrete FGF-23, which regulates phosphate metabolism by affecting the activity of Npt-2 (Sabbagh et al., 2009). FGF homologous receptors (FGFR), including FGFR 1-4, mainly bind to FGF-23 and its co-receptor α-Klotho, effectively activating FGFR 1c to form FGF-23- FGFR 1-α-Klotho ternary complex, lead to activation of mitogen-activated protein kinase (MAPK) pathway, and result in inhibition of Npt-2a and 2c in phosphorus reabsorption in renal tubular epithelial cells. It can also inhibit renal 1α-hydroxylase, reducing the serum calcitriol mediated increment of blood phosphate. Calcitriol receptor is mainly expressed in the small intestine, bone, kidney, and parathyroid gland. Its physiological effect is to increase blood phosphate and calcium. In the small intestine, calcitriol increases the absorption of phosphate by up-regulating the activity of Npt-2 in intestinal epithelial cells (Takashi and Fukumoto, 2020a). Binding of phosphorus to the calcium-sensitive receptor (CaSR) on parathyroid cells stimulates parathyroid hormone (PTH) secretion (Centeno et al., 2019). The target organs of PTH are mainly kidney and bone, and its net effect is to increase blood calcium and reduce blood phosphate. PTH promotes the reabsorption of Ca^2+^ by distal convoluted tubules and collecting ducts, reducing calcium excretion. On the other hand, PTH inhibits the reabsorption of phosphorus in proximal and distal tubules by blocking Npt-2a and 2c, promoting phosphorus excretion and reducing blood phosphate. PTH also activates 1α- hydroxylase in mitochondria of proximal renal tubular cells and facilitates the formation of calcitriol, which indirectly promotes the absorption of calcium and phosphate by intestinal epithelial cells. Moreover, PTH activates osteoclast, enhances its osteolytic effect and releases calcium and phosphorus. In osteoblasts, the binding of PTH to PTH-related protein receptor (PPR) 1 promotes FGF-23 secretion by activating protein kinase A (PKA) and Wnt signaling (Chande and Bergwitz, 2018). In addition, serum iron, erythropoietin (EPO) and insulin-like growth factor-1 (IGF-1) are also involved in the regulation of phosphate. Iron deficiency may promote FGF-23 synthesis through transcriptional regulation of hypoxia-inducible factor (HIF) 1α (Tan et al., 2017). EPO can induce the expression of FGF-23 mRNA in bone marrow erythroid cells (van Vuren et al., 2020). IGF-1 inhibits transcription factor FoxO1 of FGF-23 through phosphatidylinositol 3-kinase (PI3K)/Akt pathway, and negatively regulates FGF-23 (Bär et al., 2018).

## The Role of Phosphate in CVD

High serum phosphate level is independently associated with increased cardiovascular mortality in CKD patients by contributing to the development of CVD via distinct mechanisms (Table 1). Thus, phosphate is considered as a therapeutic target to improve CKD-associated CVD morbidity, and a detailed understanding of the molecular insight of hyperphosphatemia in the development of CVD is essential to explore effective therapeutic strategies.

**TABLE 1:** Cardiovascular pathomechanisms of hyperphosphatemia.

CVD	Underlying mechanisms
Hypertension	• Activation of SNS (Mizuno et al., 2016)
	• Increasing renin expression leading to increased circulating angiotensin levels (Bozic et al., 2014)
	• Acute impairment of endothelium-dependent vasodilation (Six et al., 2014)
	• Increase endothelin-1 production via up-regulation of aortic endothelin converting enzyme-1 expression (Olmos et al., 2017)
	• Down-regulating α-klotho expression in the kidney (Hu et al., 2015)
	• Deterioration in renal function (Da et al., 2015; Yoon et al., 2017)
Vascular calcification	• Up-regulate the expression of pit (Shobeiri et al., 2014)
	• Up-regulate osteogenic transcription of VSMCs (Singh et al., 2019; Voelkl et al., 2019; Bao et al., 2020; Chang et al., 2020; Takashi and Fukumoto, 2020b)
	• Pro-inflammatory cytokines (Voelkl et al., 2019; Alesutan et al., 2020)
	• Apoptosis and autophagy (Shroff et al., 2008; Liu et al., 2017; Ciceri et al., 2019; Voelkl et al., 2019)
	• Reduction of fetuin-A (Voelkl et al., 2019)
	• Remodeling of extracellular matrix (Voelkl et al., 2019)
	• Oxidative stress (Voelkl et al., 2019)
	•α-klotho deficiency (Singh et al., 2019)
Cardiac valve calcification	• Activation of NF-κB-AKT/ERK pathway (Li et al., 2017; Shuvy et al., 2019; Zhou et al., 2020)
	• Increased expression of pit-1 (Husseini et al., 2013)
Atherosclerosis	• Reduction of eNOS and promotes ROS in endothelial (Stevens et al., 2017; Roumeliotis et al., 2020)
	• Increase of FGF-23 and α-klotho deficiency (Hu and Moe, 2012; Mencke et al., 2015; Richter et al., 2016; Verkaik et al., 2018)
	• Endothelial cell apoptosis (Roumeliotis et al., 2020)
	• FGF-23 related dyslipidemia (Ellam and Chico, 2012)
	• Reduction of calcitriol promoting ox-LDL uptake in macrophages (Oh et al., 2009)
LVH and VF	•α-klotho independent binding of FGF-23 with FGFR4 (Faul et al., 2011; Grabner et al., 2015; Leifheit-Nestler et al., 2016)
	• Ca^2+^ dependent cardiomyocyte hypertrophy (Ca^2+^-CAMK II- IP3 pathway) (Mhatre et al., 2018)
	• FGF-23 induced ROS and RAAS (Böckmann et al., 2019; Dong et al., 2019)

### Hypertension

An increasing number of publications has revealed a detrimental role of inorganic phosphate in promoting hypertension in otherwise healthy individuals. Cross-sectional studies in patients with end-stage renal disease showed that the presence of hyperphosphatemia was significantly associated with high blood pressure (Mendes et al., 2017). Hyperphosphatemia was associated with a blunted decline in nocturnal blood pressure in hypertensive patients without CKD (Wang et al., 2018). A large prospective observational study in more than 9,000 hypertensive patients showed that elevated baseline serum phosphate is associated with poor blood pressure control over 5 years of follow-up (Patel et al., 2015). Administration of neutral NaPi (each 1 mM Na contains 0.55 mM of Pi) at the dose approximately 30 mmol of Pi per day for 11 weeks induced a significant increase in 24- h ambulatory blood pressure by 4/3 mmHg when compared to the control group treated with equivalent amounts of sodium as NaCl in combination with phosphate binder lanthanum carbonate to reduce phosphate absorption (Mohammad et al., 2018). This prospective randomized study has established a more definitive blood pressure-raising effect of inorganic phosphate in healthy young adults without hypertension or an antihypertensive drug treatment.

Animal experimental data indicate that dietary phosphate excess engages multiple mechanisms that promote hypertension. In the normotensive Sprague Dawley (SD) rats fed with a high phosphate diet (HPD) for 12 weeks, consumption of HPD induced hypertension and tachycardia in the resting condition and augments cardiovascular and sympathetic responses during muscle contraction (Mizuno et al., 2016). This result implicates that short-term dietary phosphate loading is able to transform the autonomic regulation of blood pressure in normotensive rats to the phenotype observed in hypertensive rats. The mechanisms underlying sympathetic activation induced by dietary phosphate loading are unknown. Other than the activation of the sympathetic nervous system, HPD was shown to increase renin expression, resulting in increased circulating angiotensin levels in healthy rats (Bozic et al., 2014). Acute impairment in endothelium-dependent vasodilation will happen when aortic rings are exposed to culture media with high phosphate milieu (Six et al., 2014). Increases in endothelin-1 production via upregulation of aortic endothelin-converting enzyme-1 protein expression has been demonstrated in one study in aortic endothelial cell culture upon exposure to high extracellular phosphate condition (Olmos et al., 2017). HPD has also been shown to downregulate klotho expression in the kidneys and reduce soluble klotho levels in the serum of mice (Hu et al., 2015). CKD is one of the major risk factors for the development of hypertension, and deterioration in renal function constitutes another potential mechanism by which dietary phosphate excess promotes hypertension (Da et al., 2015; Yoon et al., 2017). Large randomized clinical trials are needed to confirm the findings in animal experiments, and the results may lead to a new paradigm in preventing hypertension in the population at high risk for progression to hypertension.

### Vascular Calcification

The risk of cardiovascular death in patients with CKD stage 3a-4 is increased by 2–3 times, and the most common impairment is vascular calcification (VC) (Takashi and Fukumoto, 2020a). To date, there is no epidemiological data with a large sample to show the incidence of VC in CKD. Adeney et al. analyzed 439 patients with moderate to severe CKD and found that the incidence of calcification in the coronary artery and descending aorta increased by 21 and 33%, respectively, with the increase of blood phosphate every 1 mg/dL (Sarnak et al., 2019). The prospective cohort study, CRIC, included 1,123 patients with mild to moderate CKD (EGFR 20–70 ml/min/1.73 m^2^), and demonstrated that serum phosphate > 3.9 mg/dL was significantly associated with coronary artery calcification (Mendes et al., 2017; Bundy et al., 2018). Shang et al. (2017) selected 70 peritoneal dialysis patients without coronary artery calcification as the research objects, followed up for at least 3 years, and revealed that hyperphosphatemia was an independent risk factor for coronary artery calcification in dialysis patients. Two large cohort studies showed that serum phosphate > 3.9 mg/dL was independently associated with coronary artery calcification, even in the normal range (Patel et al., 2015). These clinical studies provide evidence that high phosphate might be a causal factor in the development of vascular calcification in CKD patients.

High phosphate is thought to contribute to the development of vascular calcification by inducing the formation of osteoblast-like cells from vascular smooth muscle cells (Cozzolino et al., 2019). Bao et al. stimulated mice with high phosphate and showed that aortic rings in high phosphate group were obviously calcified by calcium staining (Chang et al., 2020). The perception of extracellular phosphate in vascular smooth muscle is mediated by type III sodium-dependent phosphate co-transporters Pit1 and Pit2, and high phosphate up-regulates the expression of Pit in vascular smooth muscle cells (Shobeiri et al., 2014). Experimental studies indicated that activation of GK1/NF-κB, SGK1/NF-κB, Wnt/β-Catenin signaling, up-regulating osteogenic transcription factors Runx2, Msx2, Sox9 and osterix are classic mechanisms of osteogenic differentiation of vascular smooth muscle cells, among which Runx2 plays a decisive role (Voelkl et al., 2019). Pro-inflammatory cytokines are a class of endogenous peptides mainly produced by immune cells, including interleukin (IL) -1β, tumor necrosis factor (TNF) – α, IL-6. *In vitro* studies showed that IL-1β and TNF-α could stimulate the intracellular NF-κB signaling, and IL-6 activates BMP-2-wnt/β-Catenin pathway to induce the calcification of vascular smooth muscle cells (Voelkl et al., 2019; Alesutan et al., 2020). Increased FGF-23 levels and pro-inflammatory cytokines were found in patients with hyperphosphatemia (Mendoza et al., 2017). A previous prospective study, including 3,879 CKD stage 2–4 patients, indicated that FGF-23 was positively correlated with pro-inflammatory cytokines such as IL-6, CRP, and TNF-α, but there was no experiment to confirm the causal relationship between FGF-23 and pro-inflammatory cytokines (Mendoza et al., 2012). Recent studies have found that GAS5/miR-26-5p/PTEN, FGFR1c-MEK/ERK-GALNT3 pathway and α-Klotho deficiency are also important mechanisms of vascular calcification. Chang et al. showed that high phosphate stimulation downregulated the expression of a long-chain non-coding RNA (IncRNA) growth specific inhibitor GAS5 in human aortic smooth muscle cells, which weakened the inhibition of small nucleolar RNA miR-26b-5p, and thereby its downstream target protein PTEN. PTEN is a protein/lipid phosphatase, which stimulates the osteogenic differentiation of mesenchymal cells by increasing the expression of Runx2 (Bao et al., 2020; Chang et al., 2020). Takashi et al. found that high phosphate binding to FGFR1c activates downstream extracellular regulated protein kinase MEK/ERK phosphorylation and promotes the expression of osteogenic related proteins. Meanwhile, it also promotes the expression of FGF-23 post-transcriptional modified gene peptide *N*-acetylgalactosamine transferase 3 (GALNT3), which inhibited FGF-23 cleavage at multiple glycosylation sites (Takashi and Fukumoto, 2020b). Singh et al. (2019) found that aortic balloon calcification was aggravated and the expression of osteogenic proteins such as matrix metalloproteinase (MMP) 9 and 13 was up-regulated in klotho gene knockout animal models. In addition, high phosphate induces apoptosis of osteoblast-like cells by up-regulating GAS6/AXL, and releases calcium-containing apoptotic bodies (Shroff et al., 2008; Voelkl et al., 2019). Iron citrate inhibits the progression of vascular calcification through anti-apoptosis (Ciceri et al., 2019). Apoptosis is related to autophagy, and high phosphate promotes the expression of autophagy-related protein beclin-1 through the pAMPK-ULK1 pathway (Liu et al., 2017). Other mechanisms include reduction of fetuin-A (a circulating protein that inhibits calcification), remodeling of extracellular matrix and degradation of elastin, oxidative stress, lead to the progression of calcification (Singh et al., 2019; Voelkl et al., 2019).

Altogether, the clinical and experimental data emphasize that high phosphate levels may contribute to vascular calcification not only in CKD patients but also in the general population. This shows the importance of controlled serum phosphate levels and underlines that phosphate represents promising therapeutic targets in preventing vascular calcification in CKD patients.

### Valve Calcification

Valve calcification describes pathological depositions of calcium–phosphate salts on the cardiac valves. Increasing evidence has shown that mineral metabolism disorder is an important factor in promoting valve calcification (Rattazzi et al., 2013; Massera et al., 2020). The level of FGF-23 was positively correlated with the occurrence of mitral annular calcification (Bortnick et al., 2016). In another study of 6,814 CKD patients followed-up to 2.3 years, Bortnick et al. (2019) found that for every 18.5 pg/mL increase in FGF-23, the annual mitral valve calcification score measured by CT increased by 2.83 Au. The incidence of the aortic valve and mitral valve calcification increased by 25 and 62% for every 1 mg/dL increase in blood phosphate in a cohort of 439 patients with moderate CKD (Adeney et al., 2009). For every 0.5 mg/dL increase in blood phosphate, the risk of aortic valve ring calcification increases by 1.2 times (Linefsky et al., 2011). The level of blood phosphate was positively correlated with the morbidity of aortic valve calcification, and the average blood phosphate level of 3 mg/dL or more could significantly increase the risk of aortic valve calcification in another cohort of 938 subjects with clinical manifestations of chronic CVD and CKD (Hisamatsu et al., 2018).

NF-κB/Akt/ERK signaling pathway may play an important role in the high phosphate induced valve calcification. The aortic valve calcification in the HPD group was significantly higher than that in the non-phosphate control groups in the uremic rat model; Akt and ERK in the calcified valve were significantly increased; and the osteogenic transcription gene Runx-2 was overexpressed (Shuvy et al., 2019). Pit1 promotes the transfer of serum phosphate into valve interstitial cells, which is the premise of high phosphate induced valve calcification. Husseini et al. (2013) found that the expression of Pit 1 in the calcified aortic valve of human was significantly higher than that of the control group. When exposed human aortic valve interstitial cells to high phosphate medium, Runx2 will transfer from cytoplasm to nucleus, and hydrogen sulfide can reduce calcification by inhibiting Runx2 accumulation in the nucleus (Sikura et al., 2020). The osteogenic related protein Runx2 was overexpressed in the osteogenic human aortic valve stromal cells, and curcumin could suppress Runx2 gene expression by inhibiting the NF-κ B/Akt/ERK signaling pathway (Shuvy et al., 2019; Zhou et al., 2020). The expression of Runx2 was dependent on Akt and ERK phosphorylation in human aortic valve stromal cells stimulated by high phosphate (Li et al., 2017). Thus, to our current knowledge, cardiac valve calcification may share common pathogenesis with vascular calcification resulting from hyperphosphatemia in CKD patients.

### Atherosclerosis

Increasing retrospective studies have found that phosphate can promote the occurrence of atherosclerosis in coronary and peripheral arteries. A cohort study involving 7,553 subjects with near normal renal function (EGFR > 60 ml/min/1.73 m^2^) found a positive correlation between high phosphate and coronary artery atherosclerosis (Shin et al., 2012). In another study, 6,329 subjects without clinical manifestations of CKD and coronary artery disease were divided into four groups (≤3.0, 3.1 – 3.3, 3.4 – 3.7, ≥3.8 mg/dL) according to blood phosphate level, and multivariate regression analysis showed that the increase of blood phosphates level indicated the increased risk of coronary atherosclerosis (Park et al., 2020). Multiple linear regression analysis also showed that FGF-23 could increase the risk of carotid atherosclerotic plaque, which reflects systemic peripheral atherosclerosis (Rodríguez-Ortiz et al., 2020). Tuzun et al. (2018) included 54 pregnant women with gestational diabetes mellitus (GDM) and 33 healthy pregnant women, and using color to predict the degree of atherosclerosis, it was found that FGF-23 in GDM group was significantly higher than that in the control group, and the increase of FGF-23 was positively correlated with doppler ultrasound evaluated carotid intima-media thickness. In addition, FGF-23 could increase the recognition rate of the Framingham risk score for carotid atherosclerosis (Hu et al., 2017). There are gender differences in high phosphate induced atherosclerosis. Analysis of 1,687 CKD stage 3–5 non-dialysis patients from the NEFRONA study showed that phosphate levels within the normal range associated with an increased risk of subclinical atheromatosis in men, whereas this risk only increased with serum levels over the normal range in women (Martín et al., 2015). This study suggested that recommended serum phosphate levels could be different for male than for female CKD patients.

At present, the pro-atherogenic mechanism of phosphate excess has not been clarified, and it is considered that endothelial dysfunction caused by high phosphate may be the main reason for promoting lipid infiltration and atherogenesis (Hsu et al., 2010; Scialla and Wolf, 2014; Tripepi et al., 2015; Richter et al., 2016). Endothelial cell injury is the first step of atherogenesis, and endothelial nitric oxide synthase (eNOS) plays an important role in promoting endothelial dysfunction. When human peripheral vascular endothelial cells continuously exposed to high phosphate environment, the production of eNOS was significantly reduced (Ellam and Chico, 2012). High phosphate promotes reactive oxygen (ROS) production, leading to the reduction of tetrahydrobiopterin, a cofactor of eNOS coupling, and the decreasing of eNOS uncoupling nitric oxide (NO) synthesis (Roumeliotis et al., 2020). FGF-23 and Klotho also play an important role in endothelial dysfunction induced by high phosphate. When injecting FGF-23 into normal mice, the endothelium-dependent cardiac and peripheral microvascular relaxation induced by acetylcholine decreased, and the microvascular relaxation ability was restored after injection of FGF-23 antibody (Verkaik et al., 2018). FGF-23/FGFR1/klotho pathway was involved in the regulation of NO synthesis. FGF-23 combined with FGFR1 stimulated the secretion of soluble Klotho, activated Akt phosphorylation, promoted NO synthesis, up-regulated the expression of antioxidant enzymes SOD2 and CAT, and alleviated the inhibition of ROS on NO synthesis (Richter et al., 2016). CKD patients are in klotho deficiency (Hu and Moe, 2012; Mencke et al., 2015), and their NO synthesis is reduced. High phosphate also promotes endothelial cell apoptosis by activating DAXX/ERK pathway and MAPK pathway (Roumeliotis et al., 2020). FGF-23 is associated with dyslipidemia and may also promote atherosclerosis. Cohort studies have found that FGF-23 is negatively correlated with high-density lipoprotein and lipoprotein a, and positively correlated with triglycerides (Ellam and Chico, 2012). Calcitriol could inhibit the uptake of oxidized low-density lipoprotein by macrophages in diabetic patients and suppress the formation of foam cells (Oh et al., 2009). The reduction of calcitriol synthesis in hyperphosphatemia can promote the formation of foam cells. However, Shiota et al. (2011) reported significantly reduced atherosclerotic plaques under high phosphate stimulation, and the researchers attributed the underlying mechanism to the reduction of monocyte activity and the apoptosis of macrophages. This study is independent of endothelial dysfunction, and the results are still controversial.

### Left Ventricular Hypertrophy (LVH) and Myocardial Fibrosis

Studies have found that high phosphate can cause LVH, myocardial fibrosis, and increase the risk of cardiovascular death through the paracrine effect of FGF-23 (Courbebaisse and Lanske, 2018; Leifheit-Nestler and Haffner, 2018; Wang and Shapiro, 2019). The prevalence of LVH is about 40% in early-stage CKD patients and 75–80% in end-stage kidney disease patients (Vogt et al., 2019). It was found that serum phosphate > 5 mg/dL was significantly correlated with the increase of left ventricular mass index (Gallieni and Pedone, 2013). Serum level of FGF-23 was positively correlated with left ventricular mass (Kestenbaum et al., 2014). In CHS study including 2,255 subjects over 65 years old, after adjusting for demographic, cardiac and renal related risk factors, FGF-23 can be used as an independent risk factor for LVH, and the left ventricular weight increases by 6.71 g for every doubling of FGF-23 (Jovanovich et al., 2013). After adding the FGF-23 inhibitor bone matrix acidic protein DMP1 into the CKD mice, the left ventricular wall thickness in the DMP1 group was significantly reduced compared with that in the CKD group (Dussold et al., 2019).

Fibroblast growth factor-23 induced LVH mainly through the klotho independent bindings of FGF-23 with FGFR4, activating the phospholipase S γ (PLS γ)/calcineurin-dependent nuclear factor of activated T cells (NFAT) signaling pathway (Faul et al., 2011; Grabner et al., 2015). Myocardial biopsy analysis on 24 patients who died of CKD indicated that 67% of them had LVH, which was characterized by significantly expressed FGFR4, NFAT, and klotho deficiency compared with the control group (Leifheit-Nestler et al., 2016). FGF-23 and Ang II share a common mechanism in the Ca^2+^ dependent cardiomyocyte hypertrophy. In neonatal rat ventricular myocytes, FGF-23 stimulates the expression of Ang II, and Ang II induces intracellular Ca^2+^ regulated Ca^2+^/calmodulin dependent protein kinase II (CAMKII)- histone deacetylase 4 (HDAC4) activation via inositol 1,4,5-triphosphate (IP3), promoting myocardial hypertrophy (Mhatre et al., 2018). Using myocardial magnetic resonance (CMR) to detect extracellular volume (ECV) to measure the degree of cardiac fibrosis, it was found that FGF-23 was significantly positively correlated with ECV (Roy et al., 2020). A total of 39 of 51 patients with rheumatic heart disease had persistent atrial fibrillation, the expressions of FGF-23 and FGFR4 were increased in patients with atrial fibrillation, which were positively correlated with atrial fibrosis. It was speculated that FGF-23/FGFR4 pathway may play an important role in promoting atrial fibrillation by atrial fibrosis (Dong et al., 2018). FGF-23 combined with FGFR4 produced ROS, activated downstream STAT3 and Smad3 proteins, and stimulated expression of matrix metalloprotein-2 (MMP-2) in myocardial tissue of patients with atrial fibrillation, leading to myocardial fibrosis (Dong et al., 2019). Renin-angiotensin aldosterone system (RAAS) related genes were significantly expressed in a uremic rat model and positively correlated with left ventricular fibrosis. In neonatal rat ventricular myocytes, FGF-23 can induce the expression of RAAS gene and transforming growth factor – β (TGF-β), and the expression of TGF-β can be reduced by RAAS inhibitor, which indicates that FGF-23 may promote myocardial fibrosis by activating local RAAS (Böckmann et al., 2019). Current studies provide evidence that hyperphosphatemia contributes to the development of LVH. Future work has to be clarified whether phosphate can induce cardiac hypertrophy directly or only indirectly. Possible indirect mechanisms such as the phosphate mediated elevation of FGF23 or development of hypertension may contribute to the development of LVH in CKD patients.

### Interventions for Phosphate Excess

According to current knowledge, hyperphosphatemia contributes to the development of various CVD. Multidimensional phosphate-lowering therapeutic strategies targeting serum phosphate by restricting diet uptake or intestinal absorption and promoting renal excretion are pursued.

### Dietary Phosphate Restriction

Inorganic phosphate is commonly used as a flavor enhancer or preservative in the processed food, and organic phosphate naturally exists in foods rich in animal protein. Restriction of dietary phosphate intake is the elemental strategy to lower serum phosphate level. A low protein diet reduces serum FGF-23 and phosphate levels in non-dialysis and dialysis CKD patients (Shinaberger et al., 2008; Iorio et al., 2012), and the restricted consumption of inorganic phosphate additives attenuates serum phosphate levels in ESRD patients (Sullivan et al., 2009; de Fornasari and Sens, 2017). Moreover, plant-based diet usually reduced FGF23 levels but not serum phosphate levels compared with a meat-based diet (Moe et al., 2009, 2011; Scialla et al., 2012). The KDOQI guidelines recommend adjusting dietary phosphate intake to maintain serum phosphate levels in the normal range in adults with CKD 3-5D, and in adults with CKD 1-5D or post-transplantation (Ikizler et al., 2020). However, the reduction of total protein intake was associated with increased mortality, and a general restriction of protein uptake is not a suitable therapeutic strategy to reduce serum phosphate (Shinaberger et al., 2008).

The uptake of phosphate depends not only on the amount of phosphate but also on the source. The bioavailability increases from organic plant phosphate to organic animal phosphate and additives of the food industry (Noori et al., 2010). A prospective, observational study of 224 maintenance hemodialysis (MHD) patients reported that higher dietary phosphorus intake and higher dietary phosphorus to protein ratios are each associated with increased death risk in MHD patients, even after adjustments for serum phosphorus, phosphate binders and their types, and dietary protein, energy, and potassium intakes (Noori et al., 2010; Ikizler et al., 2020). The lasted KDOQI guidelines suggested that it is reasonable to consider the bioavailability of phosphate sources (e.g., animal, vegetable, additives), the ratio of phosphate (mg) to protein (g) in food should be 10–12 mg/g to maintain the balance of protein and phosphate in CKD patients when prescribing phosphate restrictive diet (Tsai et al., 2019; Ikizler et al., 2020). Reduced consumption of processed food with flavor enhancer or preservative might show beneficial clinical outcomes. Future studies need to explore if restricted phosphate uptake improves the general and cardiovascular outcome in CKD patients.

### Phosphate Binder

Phosphate binders inhibit gastrointestinal absorption of phosphate by the formation of chelating phosphate. Non-calcium phosphate binder reduces serum phosphate level but does not increase serum calcium, which potentially benefits cardiovascular. The representative drugs include sevelamer carbonate, lanthanum carbonate and iron citrate (Kalantar-Zadeh et al., 2010., Ruospo et al., 2018). Sevelamer carbonate is an anion exchange resin, which binds to phosphate in the proximal small intestine, and is similar to iron citrate in reducing serum phosphate levels and the intact FGF-23 peptide chain (St. Peter et al., 2017; Block et al., 2019a). Lanthanum carbonate dissociates in gastric acid and combines with phosphate by ionic bond to form hydrophobic compounds, which reduce serum phosphate levels and the carboxyl terminal FGF-23. Long-term intake of lanthanum has toxic side effects on liver, bone and nervous system (Liabeuf et al., 2017). A meta-analysis of 4,622 patients in 11 RCT studies showed that non-calcium phosphate binders reduced all-cause mortality by 22% and significantly reduced coronary artery calcification (Gonzalez-Parra et al., 2011). A small sample study showed that pulse wave velocity (PWV), an indicator of cardiovascular events, was significantly reduced in patients with low vascular calcification score and normal blood phosphate after 8 weeks of sevelamer hydrochloride treatment (Jamal et al., 2013). Sevelamer also has the effect of lowering blood lipid and stabilizing plaque, and significantly reduces the atherosclerotic plaque of thoracic aorta in uremic animal model with atherosclerosis (de Krijger et al., 2019). Compared with nicotinamide drugs, sevelamer reduces the low molecular endotoxin in uremic patients and reduces the chronic inflammatory reaction (Phan et al., 2005). The data from a meta-analysis included 77 studies involving 12,562 patients with CKD, showing that sevelamer significantly reduced all-cause mortality by 61% and lanthanum carbonate by 21% when compared with calcium-containing phosphate binder (Lenglet et al., 2019). Iron citrate decomposes ferric ion (Fe^3+^) and combines with phosphate. Recent studies indicate beneficial effects of ferric citrate treatment for CKD patients (Palmer et al., 2016; Block et al., 2019a,b). Besides reducing blood phosphate and inhibiting FGF-23 secretion, iron citrate increases serum iron reserve and improves anemia (Francis et al., 2019). As shown by Francis et al., ferric citrate also improves the renal and cardiac function in the Col4a3 knockout mouse model of progressive CKD. Treatment with ferric citrate reduced blood urea nitrogen levels and albuminuria, showed less renal interstitial fibrosis and tubular atrophy, and attenuated cardiac dysfunctions than Col4a3 knockout control mice. Finally, ferric citrate slowed the progression of CKD and improved the survival of CKD mice (Francis et al., 2019). If the findings of this animal study can be confirmed by clinical trials, ferric citrate and other non-calcium-phosphate binder represent promising drugs to improve cardiac and kidney function in CKD patients.

In contrast, calcium-containing phosphate binders did not lower or even increased serum FGF23 levels and promoted the progression of vascular calcification (Oliveira et al., 2010; Block et al., 2012; Jamal et al., 2013). Hence, calcium-containing phosphate binders like calcium acetate are rather inappropriate to treat CKD patients. Clinical trials analyzing the actual cardiac outcome of a phosphate binder therapy in CKD patients are still lacking.

### Other Approaches

Phosphate binders inhibited the absorption of phosphate, but also up-regulated the active phosphate transporter Npt-2b in the intestine (Isakova et al., 2015). Nicotinamide, the metabolite of vitamin B3, could reduce phosphate by inhibiting the intestinal phosphate transporter Npt-2b, which is a second-line drug for phosphate lowering treatment (Ren et al., 2020). It has been reported that nicotinamide can reduce phosphate by 12–34% in end-stage CKD dialysis patients (Vogt et al., 2019). In CKD patients on dialysis, nicotinamide might have beneficial clinical outcomes owing to the reduction of serum FGF23 and phosphate levels. However, the combination of phosphate binder and nicotinamide did not significantly reduce the levels of serum phosphate and FGF-23 in a randomized, placebo-controlled studies including 205 3b/4 CKD patients during a long-term follow-up of 12 months (Ix et al., 2019). Future studies have to consider new approaches for the long-term control of FGF23 and phosphate levels in non-dialysis CKD patients.

High serum phosphate and low serum magnesium levels correlated with an accelerated CKD progression during a median follow-up of 44 months in CKD patients (Sakaguchi et al., 2015). The cardiovascular mortality risk reduced with increasing magnesium levels in hemodialysis patients with hyperphosphatemia (Sakaguchi et al., 2014). Magnesium can inhibit the deposition of hydroxyapatite and osteogenic differentiation to prevent phosphate induced vascular calcification (Massy and Drüeke, 2015; Braake et al., 2019; Nakagawa et al., 2020). Therefore, magnesium supplementation might be a promising strategy to reduce hyperphosphatemia-associated cardiovascular risk in CKD patients. A small RCT study recruited 125 CKD stage 3–4 patients with risk factors for coronary artery calcification and followed-up for 2 years, the results showed that magnesium oxide group significantly improved vascular calcification in patients with basal coronary calcification score > 400 (Sakaguchi et al., 2019a,b). However, treatment with magnesium oxide did not influence serum phosphate levels and FGF-23 levels were not measured in this study. An animal study indicated the relevance of low magnesium diet with lower serum FGF-23 levels (Sakaguchi et al., 2019a), but FGF-23 levels were reduced after administration of calcium acetate/magnesium carbonate for 25 weeks in CALMAG study (Covic et al., 2013). There is no study report the direct connection between magnesium and CVD outcomes. Future studies should investigate if magnesium supplementation can reduce the cardiovascular mortality in CKD patients.

Promoting phosphate excretion might be another approach to control serum phosphate levels. NaPi-2b knockout uremic mice exhibit lower serum phosphate levels than wild uremic littermates (Schiavi et al., 2012). NaPi-2a inhibitor increased the excretion of phosphate in a dose-dependent manner and reduced serum phosphate levels in both healthy and uremic mice (Thomas et al., 2019). Future clinical studies will have to investigate the outcome of NaPi-2a and 2b inhibition in CKD patients. It is also conceivable that a combined treatment, which inhibits both intestinal absorption and renal reabsorption, could have the most beneficial effect for CKD patients.

## Conclusion

Hyperphosphatemia participates in the occurrence and development of a variety of cardiovascular diseases, as an important risk factor for the excessively increased cardiovascular mortality, especially in CKD population. FGF-23 plays a key role in controlling serum phosphate levels to attenuate phosphate-induced CVD. In the future, the therapeutic strategies of hyperphosphatemia need to take FGF-23 as an important target besides direct dephosphorization. The clinical compliance of dietary phosphate restriction is poor, and nutrition education should be strengthened. In different degrees of hyperphosphatemia, there is no clear definition to the target range of dephosphorization with long-term cardiovascular benefits. Promising therapeutic strategies beside dietary phosphate restriction, phosphate binders or other approaches targeting both phosphate and FGF-23 need to be further explored by large randomized clinical trials with hard endpoints to establish a causal relationship between phosphate excess and CVD, and to determine if lowering serum phosphate constitutes an effective intervention for prevention and treatment of CVD.

## Author Contributions

CZ, NO, and ZS contributed to the collection of the reference and drafted the manuscript. CZ, NO, and XR revised the manuscript. All authors contributed to the article and approved the submitted version.

## Conflict of Interest

The authors declare that the research was conducted in the absence of any commercial or financial relationships that could be construed as a potential conflict of interest.
